# Evaluating the application of the 2009 Institute of Medicine gestational weight gain guidelines on pregnant Chinese women

**DOI:** 10.1080/16549716.2023.2213494

**Published:** 2023-05-23

**Authors:** Haili Jiang, Yin Jia, Xueying Wang, Chengyan Zhang, Yue Li, Huili Wang

**Affiliations:** a Department of Obstetrics, Beijing Obstetrics and Gynecology Hospital, Beijing Maternal and Child Health Care Hospital, Capital Medical University, Beijing, China; b School of General Practice and Continuing Education, Capital Medical University, Beijing, China

**Keywords:** Gestational weight gain, body mass index, retrospective cohort study, Chinese women, perinatal outcomes

## Abstract

**Background:**

The 2009 Institute of Medicine (IOM) gestational weight gain (GWG) guidelines were initially developed for pregnant women in the United States.

**Objective:**

This study aimed to investigate whether the IOM guidelines were suitable for pregnant Chinese women.

**Methods:**

A retrospective cohort study comprising 20,593 singleton pregnant women was conducted at the Beijing Obstetrics and Gynaecology Hospital (1 January 2018 to 31 December 2019). Applicability was evaluated by comparing the GWG corresponding to the lowest point of the predicted composite risk curve with the 2009 IOM GWG Guidelines. The IOM Guidelines serve as the standard for the GWG categories and the pre-pregnancy body mass index. An exponential function model was used to fit the weight gain during pregnancy and the probability of caesarean section, preterm birth, small for gestational age, and large for gestational age. A quadratic function model was used to fit the combined probability of the above-mentioned adverse pregnancy outcomes. The applicability of the IOM guidelines was evaluated by comparing the weights corresponding to the lowest predicted probability with the GWG range recommended by the IOM guidelines.

**Results:**

According to the 2009 IOM GWG Guidelines, 43% of the women achieved adequate weight, almost 32% gained excessive weight, and 25% gained inadequate weight. The GWG range proposed by the IOM included the lowest predicted probability value for underweight women and exceeded the lowest predicted probability for normal weight, overweight, and obese women.

**Conclusions:**

The 2009 IOM guidelines were suitable for Chinese women whose pre-pregnancy body mass index was classified as underweight. The guidelines were not suitable for normal, overweight, or obese pre-pregnancy body mass index classifications. Therefore, based on the above evidence, the 2009 IOM guidelines are not suitable for all Chinese women.

## Introduction

The first 1000 days of life are extremely important in terms of nutrition for human health [1]. Weight management is crucial throughout pregnancy, and gestational weight gain (GWG), which refers to the total weight gained during pregnancy, is a vital sign of the nutritional condition of mothers and their foetuses [2]. Inadequate GWG is not only closely correlated with adverse pregnancy and neonatal outcomes, such as pregnancy-associated hypertensive disorders, post-partum weight retention, foetal macrosomia, preterm birth, and emergency caesarean sections, but it is also correlated with long-term health issues, such as hypertension, diabetes mellitus, and metabolic syndrome [3–5].

The United States Institute of Medicine (IOM) guideline, updated in 2009 [6] and based on hundreds of articles and studies, is the GWG recommendation that is internationally accepted. There is need for more primary research evidence to inform the development of country-specific guidelines. It is unclear whether the IOM guidelines can be applied to pregnant women of other nations [7–10]; the IOM GWG guidelines were mostly centred on Caucasian women. Some studies conducted in different countries and regions have evaluated the IOM guidelines regarding the incidence of adverse pregnancy outcomes. The results differ across studies, even within the same country [11–15].

There are some recent relevant studies for women in China. Yet they mostly either have small sample sizes or are not representative or comprehensive of the broader Chinese population. The question asked here is whether the 2009 IOM guidelines on weight gain during pregnancy apply to pregnant women in China.

China, a country in East Asia, is still developing and is distinct from America in terms of ethnicity, economic status, dietary habits, religious beliefs, and socioeconomic context. Asian women are often shorter and thinner than women in the United States. The pre-pregnancy body mass index (BMI) categories vary throughout Asian countries. For example, the BMI threshold for overweight and obesity in China [16] is 24 kg/m^2^–27.9 kg/m^2^ and ≥28 kg/m^2^, respectively, and in Korea [11] is 23 kg/m^2^–24.9 kg/m^2^ and ≥25 kg/m^2^, respectively, which are far lower than the World Health Organization (WHO) standards. Therefore, we assume the 2009 IOM guidelines may not be suitable for pregnant Chinese women.

Several Asian countries have examined whether the IOM guidelines were appropriate for their pregnant population. According to some studies, the 2009 IOM guidelines recommended GWG that was tolerable for the population of pregnant women [11,12]. Other studies have found that the IOM’s proposed GWG range was insufficient for pregnant women in their countries [13–15]. The two Chinese studies that have investigated the applicability of the 2009 IOM Guidelines for women in China have different results. The IOM standards were found to be appropriate for Chinese women in the study conducted in Beijing [17], whereas the study conducted in Shanghai reached the opposite conclusion [18].

Previous investigations were restricted to either small sample sizes or outdated research data. Using a large sample size, robust statistical methods, and more current research data, this study aimed to investigate whether the 2009 IOM GWG guidelines were suitable for pregnant Chinese women.

## Methods

### Study population

The study population from which the retrospective cohort was drawn comprised all singleton pregnant women who gave birth at Beijing Obstetrics and Gynaecology Hospital between January 2018 and December 2019. The Beijing Obstetrics and Gynaecology Hospital is a major facility serving the city’s 16 districts. The hospital is primarily responsible for women’s and children’s healthcare, maternal and child health education, the prevention of mother-to-child disease transmission, and family planning guidance.

Inclusion criteria were: (1) pregnant women who received regular prenatal care from conception to delivery at Beijing Obstetrics and Gynaecology Hospital, (2) healthy pregnant women with no history of cardiovascular disease, hypertension, diabetes, or haematologic diseases, and (3) pregnant women with live births, whose gestational age was at least 24 weeks. Exclusion criteria were: (1) lethal foetal malformations or stillbirths, (2) pregnant women without baseline information on height, pre-pregnancy weight, birth weight, birth length, etc., and (3) cases with clearly erroneous information.

### Data collection

The Beijing Obstetrics and Gynaecology Hospital’s medical record system provided the baseline data required for this study. First, data on the characteristics of the mothers and their foetuses (e.g. maternal age, maternal height, pre-pregnancy BMI, weight gain during pregnancy, gestational age, parity, delivery mode, neonatal birth weight, and gender), and adverse pregnancy outcomes were recorded using a structured questionnaire completed by trained midwives (Appendix 1). Logical error detection checks were made on all primary data. Where there were obvious confirmed errors, the original data were corrected. Records were deleted when the original data could not be validated. The hospital medical records system was then used to collect the research data. The study data were entered into Epidata 3.0 and exported into SPS file format for analysis.

### Anthropometric measurement

Self-reported pre-pregnancy weight in kilograms was divided by the square of height in metres to determine pre-pregnancy BMI (kg/m^2^). According to the standards of WHO [6], the maternal pre-pregnancy BMI was divided into the following four groups based on pre-pregnancy weight: underweight (BMI <18.5 kg/m^2^); normal weight (18.5 kg/m^2^ ≤ BMI <25 kg/m^2^); overweight (25 kg/m^2^ ≤ BMI <30 kg/m^2^); and obesity (BMI ≥ 30 kg/m^2^).

The midwife determined gestational age during antenatal care based on the last menstrual cycle date. Within an hour of the baby’s birth, the midwife weighed the newborn in the delivery room on the baby scale and recorded the weight in ‘g.’ Within an hour after birth, the midwife weighed the baby in the delivery room using a soft ruler and measured its length in ‘cm’.

The pre-pregnancy weight was subtracted from the last weight measured prior to labour to determine the maternal GWG. The GWG was categorised based on the 2009 IOM Guidelines [6]: 12.5–18.0 kg for underweight women (<18.5 kg/m^2^), 11.5–16.0 kg for normal weight women (18.5 kg/m^2^–24.9 kg/m^2^), 7.0–11.5 kg for overweight women (25.0 kg/m^2^–30.0 kg/m^2^), and 5.0–9.0 kg for obese women (≥30.0 kg/m^2^).

### Outcomes of interest

The outcomes that the IOM used to develop this guideline included caesarean section, preterm birth, small for gestational age (SGA), and large for gestational age (LGA).

Preterm birth was defined as giving birth before 37 full weeks of gestation [19]. For the same gestational age and sex, SGA is commonly diagnosed when the birth weight is less than the 10th percentile, and LGA is often diagnosed when the birth weight is greater than the 90th percentile [20].

### Statistical analyses

Categorical variables are expressed as frequencies and percentages; continuous variables, following the normal distribution, are expressed as means ± standard deviation. Data not meeting these criteria are presented with the median and interquartile range. Characteristics of women across GWG categories are compared using analysis of variance, the Kruskal–Wallis test (continuous variables), or the Chi-square test (categorical variables). *P* < 0.05 was considered statistically significant.

An exponential function model improved the fitbetween GWG and the predicted probability of a single adverse outcome. A quadratic function model improved the fit between GWG and the total predicted probability. The applicability was evaluated by comparing the GWG corresponding to the lowest point in the total predicted probability with the GWG of the 2009 IOM Guidelines.

The IBM statistical package for social sciences software, version 22.0, was used for data management and analysis. The predicted composite risk curve between GWG (kg) and adverse pregnancy outcomes was generated using OriginPro 2018 (version 8.5.1 SR1), with GWG (kg) as a continuous variable stratified by pre-pregnancy BMI categories.

### Ethics statement

The Medical Ethics Committee of Beijing Obstetrics and Gynaecology Hospital, Beijing, China, approved this study, which followed the Declaration of Helsinki. All participant information and records were anonymised and de-identified prior to analysis. Participants’ personal information is not publicly available.

### Results

**Figure 1.**
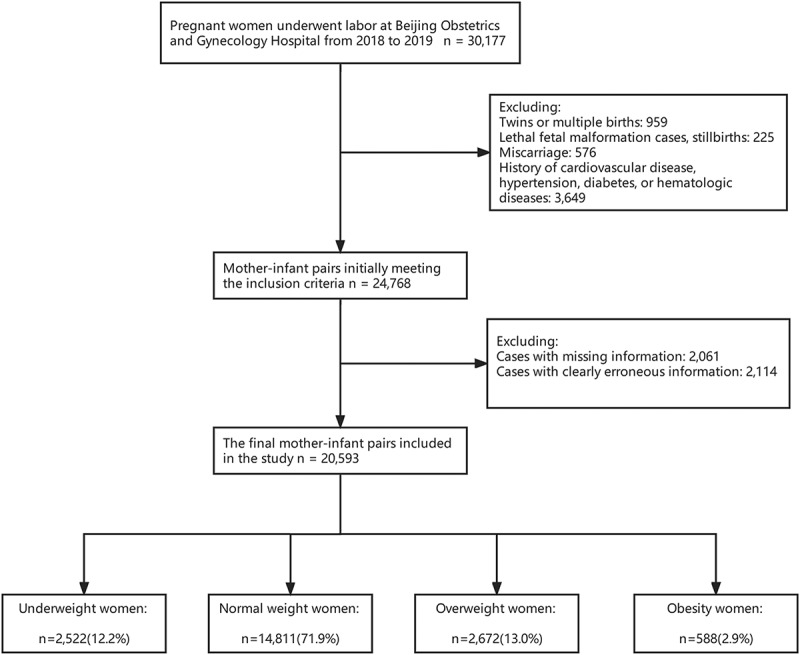
Flowchart of the inclusion and exclusion criteria of the study.

The final study cohort comprised 20,593 pregnant women who gave birth throughout the study period. Figure 1 shows the flow chart of the inclusion and exclusion of subjects.

#### Baseline characteristics of eligible pregnant women

**Figure 2.**
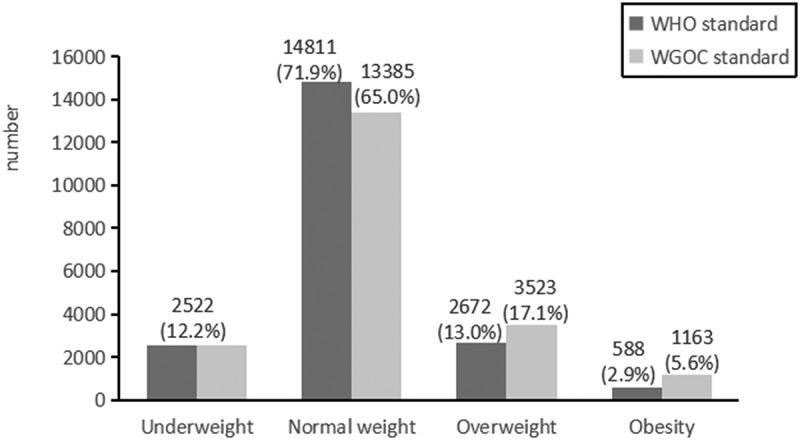
Distribution of body mass index categories before pregnancy.

**Figure 3.**
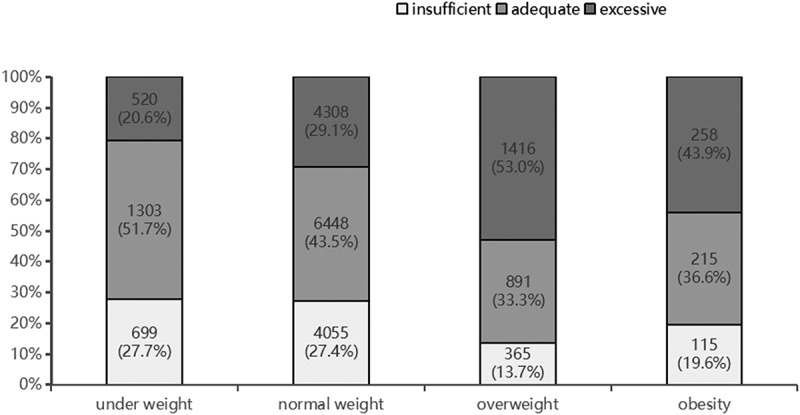
Weight gain in pregnant women under Institute of Medicine recommendations.

The mean age was 32.36 ± 3.94 years, and the mean GWG was 13.85 ± 4.99 kg among the 20,593 pregnant women. Table 1 summarises the baseline characteristics of the pregnant women and their foetuses included in this study. Figure 2 displays the distribution of pre-pregnancy BMIs of pregnant women following WHO standards and the Working Group on Obesity in China [16]. Figure 3 displays the weight gain of pregnant women based on various pre-pregnancy BMI classifications according to the IOM standard.

**Table 1.** Baseline characteristics of the included women and their infants (n=20 593).

Characteristic	
Maternal age (years), mean±SD	32.36±3.94
Maternal height (cm), mean±SD	162.84±5.01
Pre-pregnancy weight (kg), mean±SD	58.23±9.32
Gestational weight gain (kg), mean±SD	13.85±4.99
Gestational weeks, mean±SD	38.85±1.68
Pre-pregnancy BMI category n(%)	
Underweight (<18.5 kg/m^2^)	2522(12.2%)
Normal (18.5-24.9kg/m^2^)	14811(71.9%)
Overweight (25.0-29.9 kg/m^2^)	2672(13%)
Obesity (≥30.0 kg/m^2^)	588(2.9%)
Mode of delivery n(%)	
Cesarean section	6656 (32.3%)
Vaginal delivery	13937 (67.7%)
Parity n(%)	
Nulliparity	14529(70.6%)
Multiparity	6064(29.4%)
Newborn sex n(%)	
Girls	9895(48.1%)
Boys	10698(51.9%)
Gestational age classification n(%)	
SGA	1034 (5.0%)
AGA	15961 (77.5%)
LGA	3598 (17.5%)
Length of newborn (cm), mean ±SD	49.99±2.04
Weight of newborn (g), mean ±SD	3345.72±489.28

SD, standard deviation. SGA, small for gestational age. AGA, appropriate for gestational age. LGA, large for gestational age.

#### The incidence of adverse pregnancy outcomes

Only the incidence of an adequate GWG caesarean section in the underweight group was the lowest among the insufficient, adequate, and excessive weight classifications, despite the statistical significance of the incidence of caesarean section, preterm birth, SGA, and LGA in the underweight, normal weight, overweight, and obesity groups. Table 2 summarises the incidence of adverse maternal and neonatal outcomes based on the 2009 IOM GWG classification.

**Table 2.** The incidence of adverse pregnant outcomes under the standard of 2009 IOM guideline.

Prepregnancy BMI category	No.(%)	Cesarean section	Preterm birth	SGA	LGA
Underweight (n=2522)					
Insufficient	699(27.7%)	146(20.9%)**	69(9.9%)**	69(9.9%)*	54(7.7%)**
Adequate	1303(51.7%)	242(18.6%)**	33(2.5%)**	104(8%)*	110(8.4%)**
Excessive	520(20.6%)	141(27.1%)**	11(2.1%)**	27(5.2%)*	83(16%)**
Normal (n=14811)					
Insufficient	4055(27.4%)	1169(28.8%)**	320(7.9%)**	250(6.2%)**	504(12.4%)**
Adequate	6448(43.5%)	1909(29.6%)**	271(4.2%)**	285(4.4%)**	1028(15.9%)**
Excessive	4308(29.1%)	1558(36.2%)**	130(3%)**	182(4.2%)**	941(21.8%)**
Overweight (n=2672)					
Insufficient	365(13.7%)	141(38.6%)*	52(14.2%)**	23(6.3%)*	65(17.8%)**
Adequate	891(33.3%)	364(40.9%)*	71(8%)**	36(4%)*	212(23.8%)**
Excessive	1416(53%)	671(47.4%)*	93(6.6%)**	37(2.6%)*	431(30.4%)**
Obesity (n=588)					
Insufficient	115(19.6%)	51(44.3%)*	23(20%)*	5(4.3%)	26(22.6%)
Adequate	215(36.6%)	110(51.2%)*	18(8.4%)*	7(3.3%)	64(29.8%)
Excessive	258(43.9%)	154(59.7%)*	22(8.5%)*	9(3.5%)	80(31%)

GWG, gestational weight gain. BMI, body mass index. LBW, low birth weight. SGA, small for gestational age. LGA, large for gestational age. *, p<0.05; **, p<0.001

#### Evaluation of the 2009 IOM guidelines

**Figure 4.**
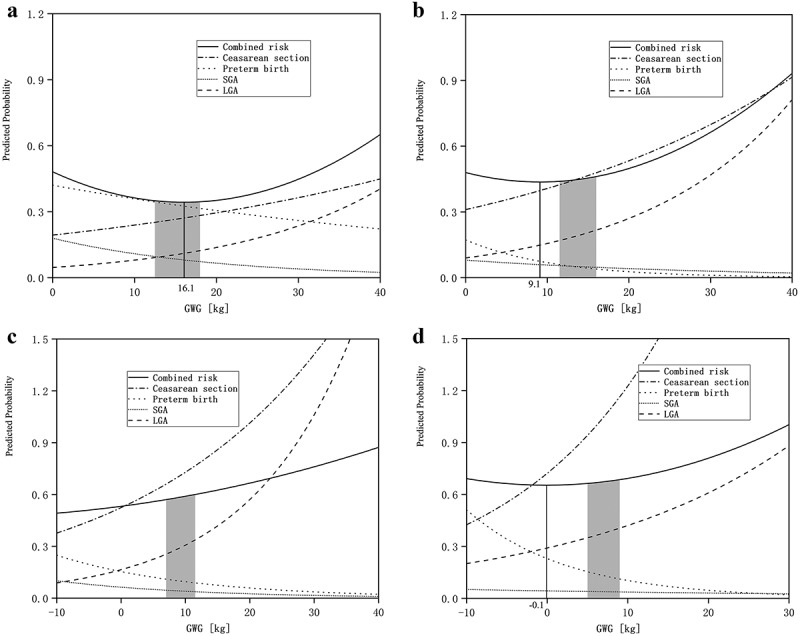
Risk curves under different body mass index categories. Underweight (a), Normal weight (b), Overweight (c), and Obese (d).

As pregnancy weight increases, the risk of caesarean section and LGA increases, while the risk of preterm birth and SGA decreases. Figure 4 depicts the association between GWG and predicted probabilities of caesarean section, preterm birth, SGA, LGA, and the combined risk of the four endpoints mentioned above (equations shown in Appendix 2).

Although the IOM recommends a GWG range of 12.5–18.0 kg, including the weight (16.1 kg), the lowest predicted probability of composite adverse pregnancy outcomes for underweight women prior to pregnancy (Figure 4(a)), for normal weight and obese women prior to pregnancy, the GWG range recommended by IOM was higher than the lowest total predicted probability (Figure 4(b,d)). For overweight women prior to pregnancy, the GWG range recommended by IOM is 7.0–11.5 kg, which is much higher than the weight (−26.9 kg, not shown in the figure) and corresponds to the lowest total predicted probability (Figure 4(c)).

## Discussion

This extensive cohort study was conducted in China to estimate the suitability of the 2009 IOM guidelines for weight gain during pregnancy. The findings indicate that the 2009 IOM guidelines could reduce the risk of adverse pregnancy and neonatal outcomes for pregnant women in China. However, the appropriate BMI weight gain range recommended by IOM during pregnancy does not include the weight gain with the lowest risk of adverse pregnancy outcome, with the exception of women classified as underweight pre-pregnancy. These results suggest that, in the Chinese context, the IOM recommended weight gain range, although high, is suitable for women who were underweight before pregnancy.

China is a country in eastern Asia. China is similar to Japan and South Korea in Eastern Asia body shape and culture. However, the findings of studies in other Asian countries on the adaptability of the IOM Guidelines vary [11,14–16]. According to some studies, pregnant women in Korea (using Korean BMI categories) and Japan can use the 2009 IOM guidelines [11,15]. Other studies conducted in Korea indicate that pregnant women in Korea do not gain weight within the low and narrow ranges recommended by the 2009 IOM guidelines [16]. According to a study conducted in Japan, pregnant Japanese women tend to gain weight at higher than recommended levels stated in the 2009 IOM guidelines [14].

Research into whether the 2009 IOM guidelines are applicable in China is contradictory. According to Yang et al. [18], the 2009 IOM guidelines could improve the prognosis of pregnant Chinese women. However, according to Song et al. [17], the range of weight gain during pregnancy recommended by the 2009 IOM guidelines could not significantly lower the risk of adverse pregnancy and neonatal outcomes. They suggested that the combined risk of low birth weight and macrosomia was lowest when the weight gain during pregnancy was less than the IOM standard.

Differences in research methodologies are one reason for inconsistent results. Many studies have similarly aimed to determine whether the recommended IOM weight gain range is appropriate by dividing pregnant women into insufficient, appropriate, and excessive weight gain groups [11,15,18]. The results generally show that weight gained in pregnancy can reduce the risk of preterm delivery but increase the risk of caesarean section, as well as LGA and SGA [21,22]. However, this approach does not determine whether pregnant women with weight gain within the appropriate range have a lower overall predicted probability of preterm birth, caesarean section, LGA, and SGA. In 2009, Beyerlein et al. [13] investigated the optimum range of weight gain during pregnancy. They used the joint predicted risk of LGA and SGA outcomes. When used to study the appropriate weight gain during pregnancy, this method can systematically analyse the overall predicted probability of various pregnancy and delivery outcomes directly associated with excessive or insufficient weight gain throughout pregnancy [12,23].

The various pre-pregnancy BMI categories may be a further reason for the different findings. Studies have demonstrated a strong correlation between pre-pregnancy BMI and maternal pregnancy and neonatal outcomes. Adverse pregnancy outcomes, which include gestational diabetes mellitus, hypertensive disorder complicating pregnancy, and foetal macrosomia, are proportional to the pre-pregnancy BMI [24,25]. The risk of SGA and premature birth is inversely proportional to pre-pregnancy BMI. Low levels of pre-pregnancy fat reserves, inadequate nutrition, and the loss of micronutrients during pregnancy contribute to adverse outcomes [22,26,27]. One of the most commonly used indicators of obesity and underweight is BMI. The IOM guidelines classify pregnant women into low weight, appropriate weight, overweight, and obese before pregnancy, based on the WHO’s BMI classification standard. The IOM guidelines also recommend the appropriate weight range for women who fall into this category, while pregnant. However, because Asians have higher body fat percentages than Caucasians, the WHO’s BMI category cannot be applied to Asian women [28]. The Asian BMI classification reference standard by the WHO was created for the Asian population in general [29]. Subsequently, several Asian countries, e.g. Vietnam and Japan, now have BMI reference standards that apply to their residents [23,30].

The Working Group on Obesity in China provided the following Chinese adult BMI classification standards [16]: underweight (BMI <18.5 kg/m^2^), normal weight (18.5 kg/m^2^ ≤ BMI <24 kg/m^2^), overweight (24 kg/m^2^ ≤ BM < 28 kg/m^2^), and obesity (BMI ≥ 28 kg/m^2^). The WHO BMI classification yields lower critical values for overweight and obesity. According to studies, a change in BMI before pregnancy alters the range of appropriate weight gain throughout pregnancy [14]. It was perhaps due to the same pre-pregnancy BMI classification (BMI <18.5 kg/m^2^) for underweight pregnant women before pregnancy that Chinese women with a pre-pregnancy underweight classification can safely gain weight in line with the 2009 IOM guidelines.

In this study, 1426 pregnant women met the WHO’s classification standard for BMI before pregnancy, which is a higher number than the BMI standard used in China. The comparison demonstrates that the baseline pre-pregnancy BMI for Chinese women classified as normal, overweight, and obese, is more generous using the WHO’s BMI classification. Therefore, the ‘permitted’ range of pregnancy weight gain for Chinese women is high according to the WHO BMI classification.

The fact that the adverse outcomes in this analysis were also used in the IOM guidelines is a major strength of this study. When assessing the applicability of the 2009 IOM GWG Guidelines, it is more scientific to consider the combined probability of the above-mentioned adverse pregnancy outcomes rather than a single outcome indicator. Based on the findings of this study, clinical obstetricians in China will be able to provide pregnant women with more individualised and personalised perinatal nutrition recommendations, while also improving their knowledge and understanding of the IOM GWG Guidelines.

This study has several limitations. Since this is a single-centre retrospective study, the findings need to be further verified. Second, outcomes, such as postpartum weight retention and long-term childhood obesity, were not investigated here. The IOM guidelines did investigate these two outcomes. We acknowledge that our results could be enhanced by extending the cohort’s follow-up period to measure postpartum weight retention and chronic obesity in children. Further, this study only used the predicted risk curve method, which could introduce selection bias. Therefore, it is important to further examine and utilise several methods to fully evaluate the applicability of IOM Guidelines.

Not only is pregnancy a crucial time for the growth and development of the embryo but it is also a crucial physiological stage for mothers [31–33]. Unbalanced nutrition can lead to either excessive or insufficient weight gain during pregnancy [34–36]. An important clinical index, such as GWG can educate and guide women towards achieving appropriate weight management to promote the health of both mother and unborn baby. However, China does not have any established GWG guidelines. More well-designed prospective investigations are needed to inform the development of guidelines to improve weight management for pregnant Chinese women.

## Conclusion

In conclusion, Chinese women who were underweight before pregnancy can safely adhere to the 2009 IOM Guidelines. However, for those Chinese women classified as having a pre-pregnancy normal, overweight, or obese BMI, the current recommended pregnancy weight gain range appears to be too high. These women could therefore be advised to gain less weight during pregnancy than stated in the 2009 IOM guidelines. The application of the IOM guidelines should be further investigated in well-designed studies with large sample sizes. The appropriate pregnancy weight gain range for Chinese women needs further research investigation.

## Paper context

The 2009 Institute of Medicine Gestational Weight Gain guidelines, originally developed for pregnant women in the United States, are commonly used worldwide. There is no current authoritative gestational weight gain range recommended for Chinese women. This study of more than 20,000 pregnant Chinese women found that these established guidelines were not suitable for all Chinese women.

## Appendix 1. Questionnaire for pregnant women at Beijing Obstetrics and Gynecology Hospital

**Table:**

**ID:□□□□□ Medical record number: ______________** Name: _______Age (years): _______Occupation: _______ Pre-pregnancy body weight (kg): _______ Maternal height (m): _______ Telephone number: _______ Last menstrual period: _______ Date of delivery: _______ Gestational age: _______ Pre-delivery body weight (kg): _______ Parity: _______ Length of the newborn (cm): ________ Weight of newborn (g): _______ Newborn sex: _______ Apgar score: 1-minute:_______5-minute: _______10-minute: _______ Amount of postpartum hemorrhage: _______ Mode of delivery: □Cesarean section □Vaginal delivery	

## Appendix 2. The exponential function models and the gestational weight gain (kg) when the combined predicted probability of adverse pregnancy outcomes was the lowest, according to pre-pregnancy Chinese-specific body mass index categories

**Table:**

Categories	Cesarean section	Preterm birth	SGA	LGA	Combined risk
Underweight	Y=exp(−1.641 + 0.021 x)	Y=exp(−0.866 – 0.016 x)	Y=exp(−1.716 –0.050 x)	Y=exp(−3.066 + 0.054x)	Y = 0.000537x^2^ – 0.017247x + 0.481669
Normal	Y=exp(−1.169 + 0.027 x)	Y=exp(−1.759 – 0.092 x)	Y=exp(−2.523 –0.033 x)	Y=exp(−2.408 + 0.055x)	Y = 0.000519x^2^ – 0.009448x + 0.479481
Overweight	Y=exp(−0.646 + 0.033 x)	Y=exp(−1.873 – 0.048 x)	Y=exp(−2.758 –0.046 x)	Y=exp(−1.801 + 0.062x)	Y = 0.000091x^2^ + 0.004888x + 0.531713
Obese	Y=exp(−0.326 + 0.053 x)	Y=exp(−1.472 – 0.080 x)	Y=exp(−3.146 –0.017 x)	Y=exp(−1.236 + 0.037x)	Y = 0.000386x^2^ + 0.000068 x + 0.653351

SGA, small for gestational age; LGA, large for gestational age.
